# Association Between Vitamin D Level and Clinical Outcomes of Assisted Reproductive Treatment: A Systematic Review and Dose-Response Meta-Analysis

**DOI:** 10.1007/s43032-024-01578-9

**Published:** 2024-05-22

**Authors:** Chenhao Xu, Xinqi An, Xiumei Tang, Yunxiao Yang, Qi Deng, Quanling Kong, Ying Hu, Dongzhi Yuan

**Affiliations:** 1 Department of Obstetrics and Gynecology, Key Laboratory of Birth Defects and Related of Women and Children of Ministry of Education, West China Second University Hospital, Sichuan University, Chengdu, Sichuan P.R. China; 2 West China School of Medicine, Sichuan University, Chengdu, Sichuan P.R. China; 3 Department of Respiratory and Critical Care Medicine, Frontiers Science Center for Disease-Related Molecular Network, West China School of Medicine, Med-X Center for Manufacturing, West China Hospital, Sichuan University, Chengdu, Sichuan P.R. China; 4 Health Management Center, West China Hospital, Institute of Hospital Management, West China Hospital, General Practice Medical Center, Sichuan University, Sichuan University, Chengdu, Sichuan P.R. China; 5 College of Medicine and Life Science, Chengdu University of Traditional Chinese Medicine, Chengdu, Sichuan P.R. China; 6 Reproductive Endocrinology and Regulation Laboratory, West China Second University Hospital, Sichuan University, Chengdu, Sichuan P.R. China; 7 West China School of Basic Medical Sciences and Forensic Sciences, Sichuan University, Chengdu, Sichuan P.R. China; 8 Department of Obstetrics and Gynecology, West China Second University Hospital, Sichuan University, Chengdu, P.R. China

**Keywords:** Vitamin D, Clinical pregnancy rate, Assisted reproduction, Threshold, Dose-response meta-analysis

## Abstract

The investigation about association between vitamin D level and clinical outcomes of assisted reproductive treatment showed various outcomes. This study aimed to review the correlation between vitamin D and outcomes of assisted reproductive treatment. The search was registered on the PROSPERO database (CRD42023458040). PubMed, Embase, Medline, ClinicalTrials.gov, and Cochrane databases were searched up to July 2023. Twenty-three observational studies were selected for meta-analysis. Comparing groups with deficient and ‘insufficient + sufficient’ vitamin D level, meta-analysis showed positive correlation between clinical pregnancy rate and vitamin D (OR 0.81, 95%CI: 0.70, 0.95, *P* = 0.0001). Comparing groups with ‘deficient + insufficient’ and sufficient vitamin D level, meta-analysis showed positive correlation between vitamin D and clinical pregnancy rate (OR 0.71, 95%CI: 0.55, 0.91, *P* = 0.006), vitamin D and live birth rate (OR 0.69, 95%CI: 0.54, 0.89, *P* = 0.003). Subgroup analysis did not show the source of high heterogeneity. No correlation was found in biochemical pregnancy rate, ongoing pregnancy rate, miscarriage rate and implantation rate. In dose-response meta-analysis, a nonlinear association was found between vitamin D levels and outcomes when levels are below approximately 24 ng/L. The study shows that vitamin D level is associated with clinical pregnancy rate and live birth rate. Low vitamin D level does not influence biochemical pregnancy rate, ongoing pregnancy rate, miscarriage rate and implantation rate. Furthermore, 24 ng/L may be a possible threshold of vitamin D concentration in assisted reproduction therapy.

**Supplementary Information:**

The online version contains supplementary material available at 10.1007/s43032-024-01578-9.

## Introduction

There has been a growing focus on the role of vitamin D in the field of reproductive medicine. In foundational research, vitamin D has demonstrated significant modulation of Anti-Mullerian hormone (AMH) signaling and follicle-stimulating hormone receptor (FSHR) gene expression [1], thus modulating follicle development. Furthermore, vitamin D has been hypothesized to exert an influence on embryo implantation [2]. Evidence supports the notion that vitamin D upregulates essential target genes, which play critical roles in endometrial development, uterine receptivity, and implantation [3].

In clinical research, inquiries into the interplay between levels of vitamin D and the clinical outcomes of assisted reproductive treatment (ART) are abundant, but the outcomes remain controversial. Several studies have showed robust association between vitamin D and the success of ART [4–8]. Contrastingly, certain investigations have yielded inconclusive results, showing no evident correlation between vitamin D levels and pregnancy outcomes [9–16]. Meanwhile, there are studies showing adverse association between vitamin D and clinical pregnancy rate (CPR), chemical pregnancy rate, ongoing pregnancy rate, miscarriage rate, implantation rate, live birth rate (LBR) or embryo quality after ART [17–19]. Thus, a systematic review and meta-analysis is needed.

The threshold of serum vitamin D remains controversial till now. Vitamin D level is classified as deficiency (25(OH)D ≤ 20 ng/ml), insufficiency (21ng/ml ≤ 25(OH)D ≤ 29 ng/ml) according to American Endocrine Society Clinical Practice Guideline [20]. However, it has been suggested that assay only be used in monitoring certain conditions. Institute of Medicine (IOM) Committee Members claimed that serum 25(OH)D ≥ 20ng/ml was replete [21]. In this meta-analysis, infertile female patients were divided into 3 groups, namely 25(OH)D sufficient (≥ 30 ng/ml or ≥ 75nmol/ml), insufficient (20-30ng/ml or 50–75 nmol/ml) and deficient (< 20 ng/ml or < 50 nmol/ml). The primary objective of this systematic review and meta-analysis is to comprehensively examine the existing literature and investigate the association between vitamin D levels and ART outcomes. Also, we hope to find out potential influential factors by subgroup analysis and determine a more proper threshold for vitamin D by employing dose-response analysis.

## Method

This systematic review was registered in the International Prospective Register of Systematic Reviews (PROSPERO; CRD42023458040) and reported in accordance with the Preferred Reporting Items for Systematic Reviews and Meta-analyses (PRISMA) Statement (For detailed information, please refer our PRISMA 2020 Checklist and MOOSE Checklist within the supplementary material).

### Search Strategy

Two investigators independently carried out a literature search across multiple databases, including PubMed, Web of Science, ClinicalTrials.gov, Embase, MEDLINE, and the Cochrane Library. The search utilized a set of predefined keywords and medical subject heading (MeSH) terms, namely [(Vitamin D) OR (25-Hydroxyvitamin D) OR (Ergocalciferols) OR (Cholecalciferol) OR (Calcitriol)] AND [(assisted reproductive technology) OR (Fertilization in Vitro) OR (Sperm Injections, Intracytoplasmic) OR (Test Tube Babies) OR (IVF) OR (ICSI)] (see Supplementary file 1). The search was limited to English-language articles published until July 2023.

### Selection Criteria

Two reviewers independently conducted the process of selection. If there was disagreement, a third reviewer would be consulted to help make the final decision through discussion among all three people. The study protocol established predefined criteria for inclusion and exclusion. Published observational studies were selected for analysis, including retrospective or prospective cohort studies, case-control studies, and cross-sectional studies. Vitamin D levels were stratified into three categories: deficient (< 20 ng/mL), insufficient (20–30 ng/mL), and sufficient (≥ 30 ng/mL). Inclusion criteria includes: (i) recruitment of women undergoing ART treatment, such as in vitro fertilization-embryo transfer (IVF-ET), intracytoplasmic sperm injection (ICSI), and preimplantation genetic testing/screening (PGT-PGS/PGD); (ii) report of vitamin D concentrations measured through either blood serum or follicular fluid tests. (iii) observational studies, and (iv) were published in English. Exclusion criteria includes: (i) reviews, conference articles, letters, animal studies or guidelines; (ii) reports related to patients participating in multicenter trials. (iii) studies with incomplete data.

### Data Extraction

Two investigators independently performed data extraction. The extracted information from all included studies encompassed the following types: authors and year of publication, type of the studies, number of patients, female age, race and country, type of ART treatment, type of oocyte used in in-vitro fertilization (IVF) cycles (autologous or donated oocytes), time of sample collection, method of vitamin D assessment, vitamin D concentration, outcomes (clinical pregnancy rate, live birth rate, ongoing pregnancy rate, miscarriage rate and implantation rate) and conclusions (Tables 1 and 2).

**Table 1:** Characteristics of trials included in the meta-analysis

Author, year	No. of patients	Female age(years)	ART treatment	Outcomes	Conclusion
Anifandis 2010	101	NA	IVF/ICSI	CPR	Higher values of vitamin D were associated with lower possibility to achieve pregnancy.
Rudick 2012	188	NA	IVF/ICSI	CPR; LBR	Vitamin D deficiency is associated with lower pregnancy rates in non-Hispanic whites, but not in Asians.
Garbedian 2013	173	NA	IVF/ICSI	LBR; IR	Women with sufficient levels of vitamin D are significantly more likely to achieve clinical pregnancy following IVF.
Rudick 2014	99	NA	IVF-ET	CPR; LBR	Non-replete vitamin D status associated with lower pregnancy rates in recipients of egg donation.
Fabris 2014	267	18–34	IVF-ET	CPR; OPR	Patients who are not vitamin D replete do not have a decreased chance of becoming pregnant with egg donation.
Paffoni 2014	480	18–42	IVF	CPR; IR	High vitamin D level was related to higher CPR.
Polyzos2014	368	18–36	IVF/ICSI	CPR; LBR	Low vitamin D level was related to lower clinical pregnancy rate and live birth rate.
Firouzabadi 2014	221	NA	IVF/ICSI	CPR; IR	No correlation was found between the serum and follicular vitamin D level and the pregnancy ratein the IVF cycle.
Franasiak 2014	529	NA	FET/ICSI	CPR; BPR; OPR	Vitamin D had no relationship with IVF outcomes.
van de Vijver A 2016	280	18–39	FET/ICSI	CPR	Vitamin D deficiency does not affect pregnancy rates in FET cycles.
Abadia 2016	100	NA	IVF/ICSI	CPR; LBR; IR	Vitamin D may be associated with higher fertilization.
Banker 2017	291	21–50	IVF/ICSI	CPR; MR	Vitamin D deficiency does not have a negative influence on IVF/ICSI outcome.
Fabris 2017	1530	Donors:18–35	IVF/ICSI	IR; CPR; MR; OPR	Bioavailable vitamin D was not related to recipients’ ongoing pregnancy rate.
Ciepiela 2018	198	18-38y	ICSI/SET	CPR; LBR; MR	Oocytes matured in FF with low 25(OH)D concentration are associated with higher pregnancy and delivery rates. Low serum vitamin D concentration is associated with higher miscarriage rates.
K Y Ko 2019	1113	NA	IVF/ICSI	CPR; CLBR; MR	The CLBR of the first IVF cycle in the vitamin D-deficient group was significantly lower compared to the non-deficient group.
Liu 2019	848	NA	FET/ICSI	CPR; LBR; MR	Among Chinese women, lower serum vitamin D levels are associated with a lower fertilization rate in IVF. However, vitamin D level was not associated with the clinical pregnancy and live birth rate following IVF.
Chu 2019	500	NA	FET/ICSI	CPR; BPR; LBR; MR	The crude live birth rate achieved in women undergoing assisted reproductive treatments are associated with serum vitamin D, although statistical significance is lost when adjusting for important prognostic variables.
Walz 2020	287	NA	IVF/ICSI	CPR; BPR; MR	There was no association between Vit D and clinical pregnancy or live birth outcomes.
Cai 2021	2569	18–39	IVF/ICSI	BPR; IR; CPR; MR; OPR	The early pregnancy outcomes were similar in women with adequate, insufficient and deficient total 25(OH)D serum concentrations.
Neysanian 2021	150	18–40	IVF/ICSI	BPR; CPR	Women with higher levels of vitamin D in their serum and follicular fluid are significantly more likely to achieve pregnancy but without affecting the quality of embryo and fertility rate.
Muyayalo 2022	132	< 35	IVF/ICSI	IR; CPR; MR; LBR	VD levels in FF, but not in serum, were associated with embryo quality, normal fertilization, IRs, and clinical pregnancy rates.
Yu 2022	612	NA	IVF/ICSI	CPR; LBR	The serum level of 25(OH)D demonstrated a nonlinear positive correlation with pregnancy outcomes with stronger correlations above 25 ng/ml and worse yields below 30 ng/ml.
Hasan 2023	218	18–39	IVF/ICSI	CPR	Preconception 25(OH)D sufficiency (>50 nmol/L) is associated with successful pregnancy outcome following IVF therapy.

NA, not applicable; IVF, in vitro fertilization; ICSI, intracytoplasmic sperm injection; ET, embryo transfer; CPR, clinical pregnancy rate; LBR, live birth rate; IR, implantation rate; OPR, ongoing pregnancy rate; BPR, biochemical pregnancy rate; MR, miscarriage rate; y, year

**Table 2:** Characteristics of trials included in the subgroup meta-analysis

Author ,year	Type of studies	Race/ Country	ART treatment	Oocyte origin	Ovarian stimulation	Time ofsample collection	Method of vitamin D assessment
Anifandis 2010	PCS	NA	IVF/ICSI	AO	Short GnRH-a	During oocyte retrieval	ECLIA
Rudick 2012	RCS	Non-Hispanic Whites, Hispanic Whites, Asians	IVF/ICSI	AO	LongGnRH-a, GnRH-ant	The day after hCG administration	ELISA
Garbedian 2013	PCS	White; Black; Other	IVF/ICSI	AO	Long GnRH-a, GnRH-ant	Within 1 week before oocyte retrieval	NA
Rudick 2014	RCS	Caucasian, Asian, Hispanic, African, American	IVF-ET	DO	Long GnRH-a, GnRH-ant	At the time of down-regulation	ELISA
Fabris 2014	RCS	NA	IVF-ET	DO	GnRH-a	After 2 weeks of hormone replacement therapy	ELISA
Paffoni 2014	CS	Italy	IVF	AO	Long GnRH-a; GnRH-ant	Prior to to initiation of COH	ECLIA
Polyzos2014	RCS	Belgium	IVF/ICSI	AO	GnRH-a, GnRH-ant	On the day of hCG administration	ELISA
Firouzabadi 2014	PCS	Iran	IVF/ICSI	AO	long GnRH-a	On the dayof ovum pick-up	ELISA
Franasiak 2014	RCS	Canada	FET/ICSI	AO	Long GnRH-a, GnRH-ant	On the day of ovulation trigger	ELISA
van de Vijver A 2016	PCS	Belgium	FET/ICSI	AO	GnRH-a	On the day of embryo transfer	NA
Abadia 2016	PCS	White/Caucasian; Other	IVF/ICSI	AO	GnRH-a, GnRH-ant	Betweendays 3 and 9 of hCG treatment	ELISA
Banker 2017	PCS	NA	IVF/ICSI	DO	GnRH-a, GnRH-ant	NA	CMIA
Fabris 2017	RCS	Spain	IVF/ICSI	DO	GnRH-a, GnRH-ant	NA	ELISA
Ciepiela 2018	PCS	Poland	ICSI/SET	AO	GnRH-a, GnRH-ant	On the day of oocyte retrieval	CLIA
K Y Ko 2019	RCS	Hong Kong	IVF/ICSI	AO	Long GnRH-a, GnRH-ant	in the early follicular phase of the cycle	LC-MS
Liu 2019	RCS	China	FET/ICSI	AO	GnRH-a, GnRH-ant	on the oocyte retrieval day	CMIA
Chu 2019	PCS	White, South Asian, Black, Chinese, Other	FET/ICSI	AO	Short/long GnRH-ant	NA	LC-MS
Walz 2020	CS	Australia	IVF/ICSI	AO	GnRH-a, GnRH-ant	On the day of oocyte retrieval	CLIA
Cai 2021	PCS	China	IVF/ICSI	AO	Short/long/ultralong GnRH-a	1 day before embryo transfer	ELISA
Neysanian 2021	PCS	Iran	IVF/ICSI	AO	Long GnRH-a	On the same day of ovum pick up	LC-MS
Muyayalo 2022	PCS	China	IVF/ICSI	AO	Long GnRH-a	On the same day of ovum pick up	ECLIA
Yu 2022	RCS	China	IVF/ICSI	AO	GnRH-a, hCG	Before IVF/ICSI-ET	LC–MS
Hasan 2023	RCS	NA	IVF/ICSI	AO	GnRH-a, hCG	NA	ECLIA

NA, not applicable; PCS, prospective cohort study; RCS, retrospective cohort study; CS, cross-sectional study; IVF, in vitro fertilization; ICSI, intracytoplasmic sperm injection; ET, embryo transfer; CPR, clinical pregnancy rate; LBR, live birth rate; IR, implantation rate; OPR, ongoing pregnancy rate; BPR, biochemical pregnancy rate; MR, miscarriage rate; y, year, GnRH-a, GnRH-agonist; GnRH-ant, GnRH-antagonist; hCG, human chorionic gonadotropin; ELISA, enzyme-linked immunosorbent assay; ECLIA, electrochemiluminescence immunoassay; LC-MS, liquid chromatograph-mass spectrometer; CLIA, chemiluminescence immunoassay; CMIA, chemiluminescent microparticle immunoassay

### Quality Assessment

The evaluation of the included studies’ quality was executed utilizing the Newcastle-Ottawa Scale (NOS) independently by two reviewers.

### Statistical Analysis

We utilized Review Manager 5.3 and Stata 17.0 to conduct all statistical analyses. Heterogeneity among studies was assessed using both chi square and the heterogeneity index, in instances where significant heterogeneity was observed (I^2^ > 50%), we adopted the random effects model. Conversely, when heterogeneity was minimal (I^2^ ≤ 50%), the fixed effects model was employed. We conducted a sensitivity analysis to identify any studies that may have had an outsized impact on the overall pooled results. To assess publication bias, we employed funnel plot asymmetry and Egger’s test. Subgroup analyses were performed to isolate potential confounding factors and included the source of oocyte, source of vitamin D, race, study design, method of vitamin D assessment, and time of sample collection.

### Dose-Response Meta-Analysis

In August 2017, the Methodology Group introduced a groundbreaking meta-regression method known as the Robust-error meta-regression (REMR) for synthesizing dose-response data. We employed this novel method to conduct the dose-response analysis. We calculated study-specific slopes (representing linear trends) and their corresponding 95% confidence intervals by employing natural logarithms of the odds ratios and confidence intervals. This analysis was conducted across various categories of vitamin D levels [22].

The method necessitated the availability of case distributions or non-case and odds ratios, along with their corresponding variance estimates, for a minimum of three quantitative usage categories. Studies that categorize patients into two groups based on their vitamin D levels were excluded from the dose-response meta-analysis.

Some original studies presented exposure ranges without indicating the average or median levels of exposure. To address this, we adopted the following estimation approach: for closed intervals, we determined the exposure level as the midpoint between the upper and lower limits; for open intervals (where either the upper or lower endpoint was provided), we treated the interval length of the adjacent group as the interval length of the target group and calculated the midpoint as the estimated average exposure level [23, 24].

## Results

### Literature Selection

**Fig. 1:**
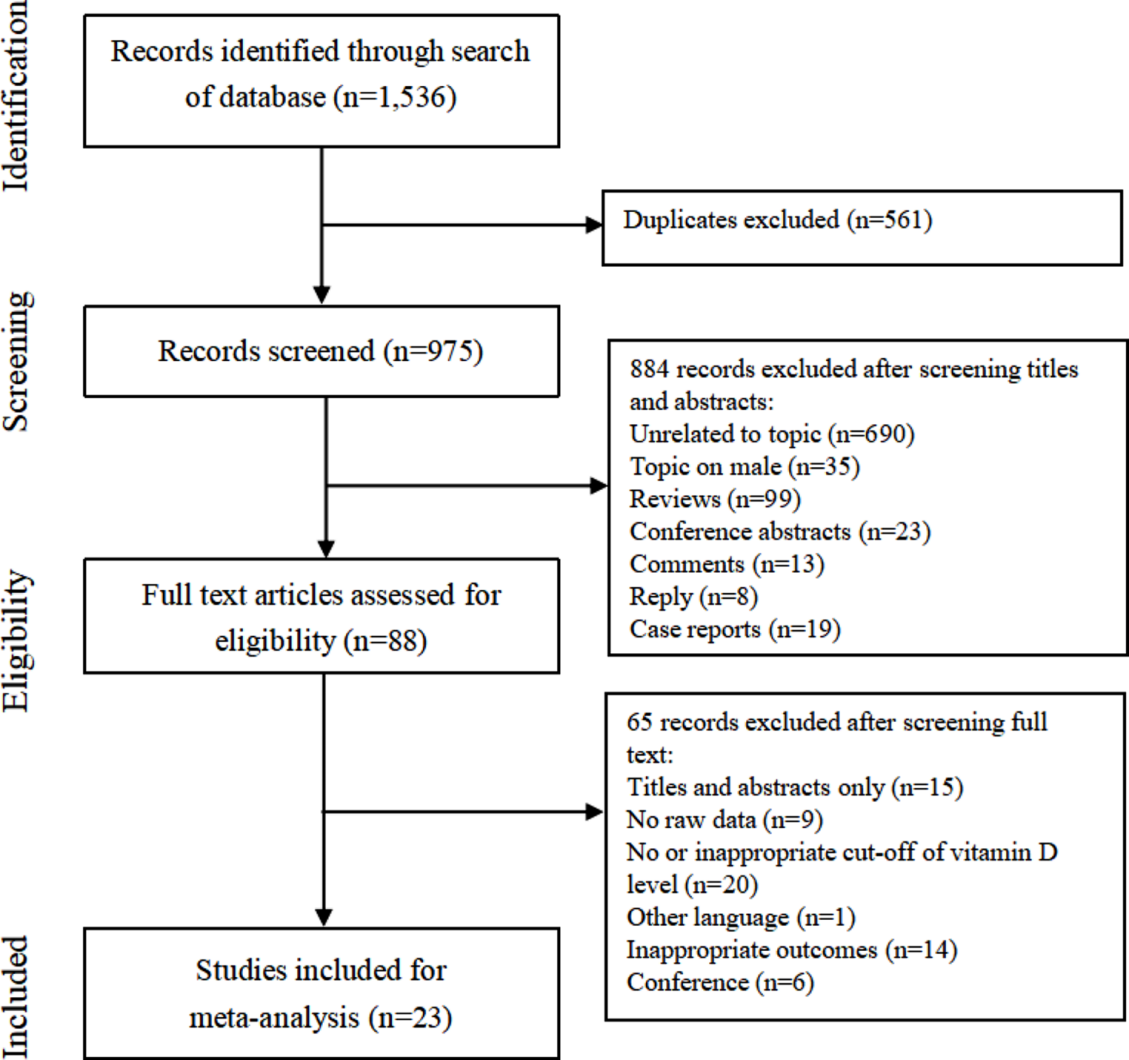
Flow chart of selection process

The electronic database search produced a total of 1,536 citations. Twenty-three [4–10, 13–19, 25–33] studies were included for meta-analysis. The PRISMA flow diagram of the review process is shown in Fig. 1.

### Description of Studies and Participants

Table 1 demonstrates a summary of the main characteristics. Table 2 presents the extracted information pertaining to subgroup analysis from the original studies.

### Quality of Studies

Newcastle–Ottawa Quality Assessment Scale (NOS) was used to evaluate the quality of the twelve studies. Studies were regarded as ‘high quality’ if they had a score > 5. Thirteen studies achieved a score of 6. Ten studies achieved a score between 7 and 8 (Supplementary file 2).

### Clinical Pregnancy Rate

#### Deficient Vitamin D Level Versus ‘Insufficient + Sufficient’ Vitamin D Level

**Fig. 2:**
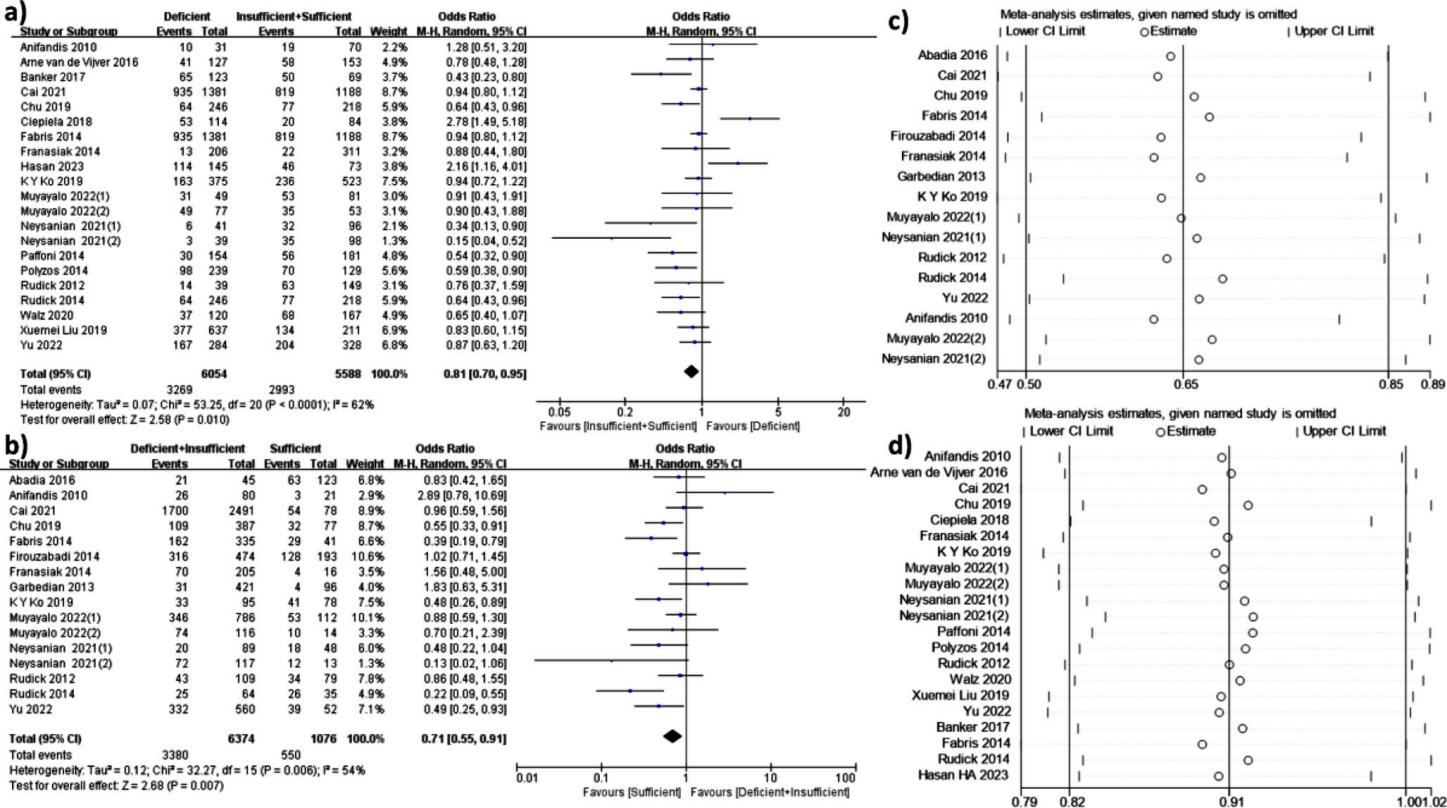
**(a)** Meta-analysis of studies reporting CPR in sufficient vitamin D(≥ 30ng/ml) + insufficient vitamin D(20-30ng/ml) and deficient vitamin D(< 20ng/ml). (**b)** Meta-analysis of studies reporting CPR in sufficient vitamin D(≥ 30ng/ml) and deficient vitamin D(< 20ng/ml) + insufficient vitamin D(20-30ng/ml). (**c)** Sensitivity analysis on Deficient vitamin D level versus ‘Insufficient + Sufficient’ vitamin D level. **(d)** Sensitivity analysis on ‘Deficient + Insufficient’ vitamin D level versus Sufficient vitamin D level. Note: (1) refers to the data of follicular vitamin D, (2) refers to the data of serous vitamin D

21 studies investigated the association between clinical pregnancy rate and vitamin D levels (Fig. 2C). The comparison was between ‘Deficient’ vitamin D level and ‘Insufficient + Sufficient’ vitamin D level. 5,588 infertile patients had sufficient or insufficient vitamin D, 6,054 had deficient vitamin D. Meta-analysis showed positive correlation between CPR and vitamin D (OR 0.81, 95%CI: 0.70, 0.95, *P* = 0.01). Since I^2^ > 50%, subgroup analyses were conducted.

To detect the origin of the heterogeneity, six subgroup analyses were conducted, including source of oocyte, source of vitamin D, race, study design, method of vitamin D assessment and time of sample collection (Table 3). The detailed forest plot results of the subgroup analyses can be found in Supplementary file 5.

**Table 3:** Subgroup analysis of studies reporting CPR in sufficient vitamin D(≥ 30ng/ml) + insufficient vitamin D(20-30ng/ml) and deficient vitamin D(< 20ng/ml)

	NO	OR(95%Cl)	*P* within group	heterogeneity		
				*P* heterogeneity	I^2^	*P* between subgroups
Subgroup analysis						
Source of oocyte	20					
AO	17	0.80(0.67,0.95)	0.01	0.002	56%	0.53
DO	3	0.69(0.45,1.06)	0.09	0.02	75%	
Source of vitamin D	20					
Serum	17	0.79(0.67,0.93)	0.006	0.002	57%	0.65
Follicular fluid	3	0.61(0.20,1.84)	0.38	0.02	76%	
Race	20					
Asian	7	0.93(0.82,1.04)	< 0.00001	< 0.0001	71%	0.004
Non-Asian	13	0.69(0.58,0.81)	0.20	0.44	0	
Study design	20					
Prospective cohort	10	0.86(0.76,0.98)	0.03	0.0001	73%	0.12
Retrospective cohort	8	0.87(0.78,0.98)	0.02	0.38	6%	
Cross-sectional	2	0.59(0.42, 0.85)	0.004	0.61	0	
Method of vitamin D assessment	18					
ELISA	6	0.89(0.80,0.99)	0.03	0.2	31%	0.14
ECLIA	3	0.69(0.48,0.99)	0.05	0.38	0	
LC-MS	5	0.77(0.65,0.92)	0.004	0.01	68%	
CLIA	2	1.16(0.801.68)	0.44	0.0003	92%	
CMIA	2	0.72(0.54,0.96)	0.03	0.06	71%	
Time of sample collection	16					
Before oocyte retrieval	6	0.80(0.68,0.94)	0.007	0.31	16%	0.42
During oocyte retrieval	8	0.84(0.69,1.03)	0.10	0.0004	74%	
After oocyte retrieval	2	0.93(0.79,1.08)	0.34	0.48	0	

‘-’ means the relevant data is not applicable

AO, autologous oocyte; DO, donor oocyte; ELISA, enzyme-linked immunosorbent assay; ECLIA, electrochemiluminescence immunoassay; LC-MS, liquid chromatograph-mass spectrometer; CLIA, chemiluminescence immunoassay; CMIA, chemiluminescent microparticle immunoassay

#### ‘Deficient + Insufficient’ Vitamin D Level Versus Sufficient Vitamin D Level

16 studies (7,450 participants) investigated the association between clinical pregnancy rate and serum vitamin D levels (Fig. 2b). The comparison was between ‘Deficient + Insufficient’ vitamin D level and ‘Sufficient’ vitamin D level. 1,076 infertile patients had sufficient vitamin D, 6,374 had deficient or insufficient vitamin D. Meta-analysis showed positive correlation between CPR and vitamin D (OR 0.71, 95%CI: 0.55, 0.91, *P* = 0.007). Since I^2^ > 50%, subgroup analyses were conducted.

To detect the origin of the heterogeneity, six subgroup analyses were conducted, including source of oocyte, source of vitamin D, race, study design, method of vitamin D assessment and time of sample collection (Table 4). The detailed forest plot results of the subgroup analysis can be found in Supplementary file 5.

**Table 4:** Subgroup analysis of studies reporting CPR in sufficient vitamin D(≥ 30ng/ml) and insufficient vitamin D(20-30ng/ml) + deficient vitamin D(< 20ng/ml)

	NO	OR(95%Cl)	*P* within group	heterogeneity		
				*P* heterogeneity	I^2^	*P* between subgroups
Subgroup analysis						
Source of oocyte	16					
AO	14	0.72(0.56,0.93)	0.01	0.04	43%	0.44
DO	2	0.39 (0.24,0.64)	0.0002	1	0	
Source of vitamin D	16					
Serum	13	0.68(0.57, 0.81)	< 0.0001	0.04	44%	0.03
Follicular fluid	3	0.54(0.31,0.95)	0.03	0.009	79%	
Race	17					
Asian	6	0.75(0.59, 0.94)	0.01	0.2	32%	0.28
Non-Asian	11	0.63(0.50,0.78)	< 0.0001	0.009	58%	
Study design	16					
Prospective cohort	10	0.67(0.53,0.84)	0.0005	0.05	46%	0.96
Retrospective cohort	6	0.67(0.53, 0.86)	0.001	0.01	66%	
Cross-sectional	0	-	-	-	-	
Method of vitamin D assessment	14					
ELISA	6	0.85(0.65, 1.12)	0.26	0.16	37%	0.25
ECLIA	3	0.68(0.40, 1.18)	0.17	0.02	74%	
LC-MS	5	0.62(0.48, 0.80)	0.0003	0.19	35%	
CLIA	0	-	-	-	-	
CMIA	0	-	-	-	-	
Time of sample collection	13					
Before oocyte retrieval	6	0.76(0.60, 0.96)	0.02	0.20	32%	0.40
During oocyte retrieval	6	0.62(0.42, 0.92)	0.02	0.03	60%	
After oocyte retrieval	1	0.96(0.59, 1.56)	0.85	-	-	

‘-’ means the relevant data is not applicable

AO, autologous oocyte; DO, donor oocyte; ELISA, enzyme-linked immunosorbent assay; ECLIA, electrochemiluminescence immunoassay; LC-MS, liquid chromatograph-mass spectrometer; CLIA, chemiluminescence immunoassay; CMIA, chemiluminescent microparticle immunoassay

### Secondary Outcomes

In comparison between Deficient vitamin D level versus ‘Insufficient + Sufficient’ vitamin D level, no correlation was found in biochemical pregnancy rate, ongoing pregnancy rate, miscarriage rate, live birth rate, implantation rate. Specific information is provided in Supplementary file 3.

In comparison between ‘Deficient + Insufficient’ vitamin D level versus Sufficient vitamin D level, no correlation was found in biochemical pregnancy rate, ongoing pregnancy rate, miscarriage rate, implantation rate. However, a positive correlation was found (OR 0.69, 95%CI: 0.54, 0.89, *P* = 0.003) between vitamin D level and live birth rate, with a relatively low I^2^. Specific information is provided in Supplementary file 3.

### Sensitivity Analysis

The outcomes of the sensitivity analysis are visually depicted in Fig. 2c and d. Notably, the removal of each individual study did not exert a substantial influence on the pooled odds ratios (ORs).

### Publication Bias

Supplementary file 4 showed the funnel plot was relatively asymmetrical. However, Egger’s test (*P* = 0.367, *P* = 0.035) indicated no publication bias, with *P* = 0.367 in the first comparison between groups with ‘deficient + insufficient’ and sufficient vitamin D level, and *P* = 0.035 in the second comparison between groups with deficient and ‘insufficient + sufficient’ vitamin D level.

### Dose-Response Analysis

**Fig. 3:**
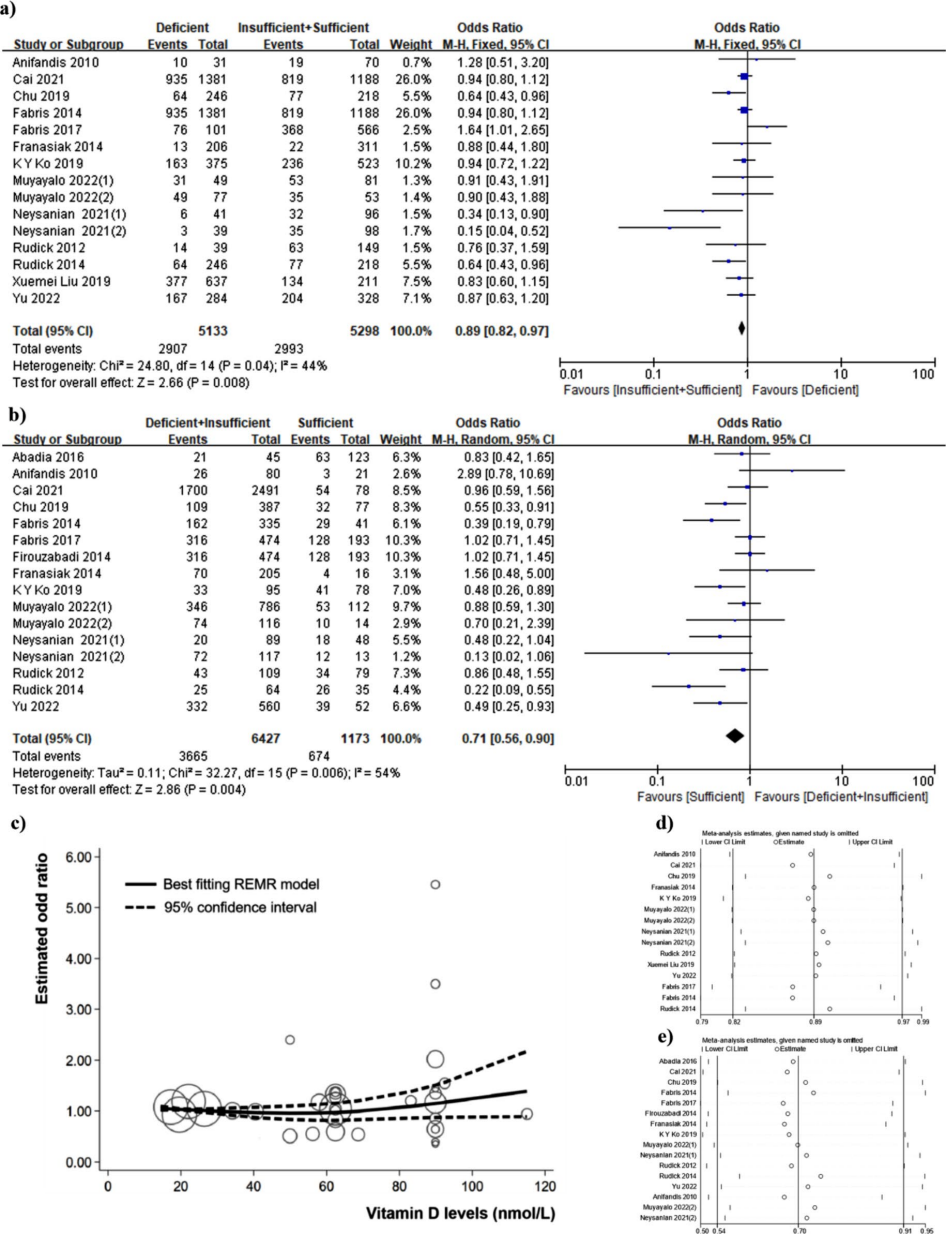
**(a)** Meta-analysis of studies reporting CPR in sufficient vitamin D(≥ 30ng/ml) + insufficient vitamin D(20-30ng/ml) and deficient vitamin D(< 20ng/ml) among selected 15 studies; (**b)** Meta-analysis of studies reporting CPR in sufficient vitamin D(≥ 30ng/ml) and deficient vitamin D(< 20ng/ml) + insufficient vitamin D(20-30ng/ml) among selected 15 studies (**c)** Dose-response analyses between vitamin D levels and CPR. Hollow circles indicate logarithms of the odd ratios with size proportional to inverse of their variance; (**d)** Sensitivity analysis on Deficient vitamin D level versus Insufficient + Sufficient vitamin D level; (**e)** Sensitivity analysis on Deficient + Insufficient vitamin D level versus Sufficient vitamin D level. Note: (1) refers to the data of follicular vitamin D, (2) refers to the data of serous vitamin D

Fifteen studies were included in the dose-response analysis, with 4283 cases among 7886 participants. The summary odds ratio was 0.89 (95%CI: 0.82,0.97) comparing ‘Deficient + Insufficient’ and ‘Sufficient’, with moderate heterogeneity (I^2^ = 44%, *P* = 0.04) (Fig. 3b). Publication bias was not evident in Egger’s test (*P* = 0.42) or Begg’s test (*P* = 0.30). In sensitivity analysis excluding one study at one time, the summary of odds ratio ranged from 0.88 (95%CI: 0.81, 0.97) when Fabris, 2014 was excluded to 0.88 (95%CI: 0.79, 0.97) when Rudick, 2014 or Chu, 2019was excluded (Fig. 3d). The summary odds ratio was 0.71 (95%CI: 0.56, 0.90) comparing ‘Deficient’ and ‘Insufficient + Sufficient’, with moderate heterogeneity (I^2^ = 54%, *P* = 0.008) (Fig. 3a). Publication bias was not evident in Egger’s test (*P* = 0.36) or Begg’s test (*P* = 0.39). The summary of odds ratio ranged from 0.67 (95%CI: 0.52, 0.86) when Anifandis, 2010 was excluded to 0.74 (95%CI: 0.58, 0.95) when Rudick, 2014 was excluded in sensitivity analysis (Fig. 3e).

When the level of vitamin D is below approximately 60 nmol/L (24 ng/L), a clear non-linear association exists between the clinical pregnancy rate and dosage. However, as the vitamin D level exceeds approximately 24 ng/L, there is a slight improvement in the linear correlation between the two variables, and a modest increase in the degree of linear correlation with higher dosage can be observed (Fig. 3c).

## Discussion

This systematic review and meta-analysis, comprising 23 individual studies, presents compelling evidence suggesting a potential link between serum vitamin D levels and the outcomes of ART. The results obtained from our analysis indicate that women who maintain sufficient vitamin D levels tend to exhibit increased probabilities of achieving successful live births, as well as positive pregnancy tests and clinical pregnancies, when undergoing ART procedures. Conversely, those individuals with deficient or insufficient levels of vitamin D demonstrate comparatively lower chances of achieving these favorable outcomes. However, there was no observed association between vitamin D levels and miscarriage rate, ongoing pregnancy rate, or biochemical pregnancy rate. Performing a sensitivity analysis is crucial in the meta-analysis of observational studies as it allows researchers to evaluate the stability and credibility of the combined point estimate [34]. The exclusion of any single study in turn did not result in substantial changes to the pooled ORs. This demonstrates the robustness of our research findings.

In the past few years, there have been five distinct meta-analyses published, examining the impact of vitamin D on the outcomes of IVF/ICSI, with varying and inconclusive findings [27, 35–38]. Compared to previous studies, our study has several advantages. (i) It includes the largest number of participants and the most extensive dataset among all published research in this field. (ii) Our sensitivity analysis yielded highly favorable results, which was not achieved in earlier studies. (iii) We conducted six subgroup analyses to identify potential sources of clinical heterogeneity in the relationship between vitamin D levels and ART outcomes. (iv) we introduced a novel approach by conducting a dose-response analysis, providing a new perspective on the issue of vitamin D threshold levels.

We performed 6 subgroup analyses to detect the origin of the heterogeneity of the clinical pregnancy rate, which is source of vitamin D, source of oocyte, race, study design, method of vitamin D assessment and time of sample collection. Unfortunately, none of these six subgroup analyses showed satisfactory heterogeneity test results. Nevertheless, some of the analyses still provided suggestive findings.

Muyayalo et al. measured the concentrations of vitamin D in both serum and follicular fluid concurrently, and examined their respective correlations with ART treatment outcomes [32]. They found that vitamin D levels in follicular fluid (FF), but not in serum, were associated with it. The observed result may be attributed to the potential antioxidant effect of vitamin D in human female gametes. It is possible that this effect is more pronounced within the follicular fluid compared to the bloodstream. Higher total antioxidant concentrations are linked to enhanced ovarian stimulation efficiency and pregnancy success in infertile patients [39]. Most studies did not differentiate between the concentrations of vitamin D in serum and follicular fluid, resulting in only three articles [5, 17, 32] being included in our subgroup analysis specifically focusing on follicular fluid. Most studies consistently show a positive link between serum and follicular fluid vitamin D levels, indicating that assessing vitamin D status in peripheral blood can reliably indicate its availability in the ovaries [9, 28, 32, 40–45].

Although there is clinical heterogeneity in the detection methods of vitamin D, our subgroup analysis did not reveal this result. Within-group heterogeneity only decreased in the ELISA and LC-MS group, with no significant statistical differences observed between groups.

According to a recent study, the administration of vitamin D supplements has been shown to enhance the survival and growth of antral follicles in addition to promoting oocyte maturation [46, 47]. Furthermore, vitamin D has been hypothesized to enhance endometrial receptivity and exert an influence on embryo implantation [2]. Evidence supports the notion that vitamin D, upon binding to its receptor (VDR), upregulates essential target genes, including HoxA10, which plays a critical role in endometrial development, uterine receptivity, and implantation [3]. Besides, sufficient vitamin D status is vital to ensure optimal gestation and maintain a healthy pregnancy [48].

The Endocrine Society defines vitamin D deficiency as serum calcitriol levels below 50 nmol/l (< 20 ng/ml), insufficiency as 50–75 nmol/l (21–29 ng/ml), and sufficiency as above 75 nmol/l (30 ng/ml). The Institute of Medicine defines deficiency as below 30 nmol/l (< 12 ng/ml), insufficiency as 30–50 nmol/l (12–20 ng/ml), and sufficiency as above 50 nmol/l (> 20 ng/ml). Besides, in the original studies included in our analysis, different cutoff points were used for vitamin D levels. Furthermore, the number of groups created based on vitamin D levels varied among studies, ranging from two to four or more groups. It is important to note that analyzing the data using only a single cutoff point may obscure potential effects of these thresholds on the outcomes. Consequently, utilizing a dose-response analysis offers a more thorough and reliable research methodology for exploring the connection between vitamin D levels and clinical pregnancy rate.

A recent study [49] carried out by Yu et al. at a hospital in China has demonstrated that there exists a non-linear connection between vitamin D levels and favorable pregnancy outcomes in women receiving ART when their vitamin D levels are below 25 ng/ml (approximately 79.5 nmol/L). The correlation becomes stronger when the vitamin D level exceeds 25 ng/ml. In our dose-response analysis, we integrated data from a total of 15 original studies, comprising reported intra-group mean values of vitamin D levels and estimated mean values obtained using scientific methods. The results of our study presented similarities to those of Yu et al. A nonlinear relationship was observed between vitamin D levels and outcome measures when levels were below approximately 24 ng/L. Specifically, below this threshold, vitamin D levels showed little impact on the pooled OR. However, beyond this threshold, the degree of correlation increased, indicating that higher levels of vitamin D directly contributed to an increase in the pooled effect size, as measured by OR.

Several studies have suggested the existence of biological thresholds for 25(OH)D conversion, below which sufficient conversion to 1,25-dihydroxyvitamin D may not be maintained [50]. We postulate that, from a clinical perspective, it is possible that minimal variations in vitamin D levels, whether slightly higher or lower than a very low threshold, do not yield qualitative changes and have no significant impact on pregnancy rates. This observation aligns with the previously mentioned conversion process of vitamin D within the body. In terms of statistical analysis, if the women included in the study predominantly exhibit vitamin D levels below a certain threshold (approximately 24 ng/L according to this study; approximately 25 ng/ml according to Yu et al.), or if appropriate grouping has not been conducted, it becomes challenging to observe intergroup differences. Thus, establishing a direct link between vitamin D levels and the likelihood of pregnancy may be hampered.

Certainly, our research does have some limitations. First, the dose-response analysis conducted in our study has revealed substantial challenges that warrant careful consideration. Many of the original studies included in this research have missing values for vitamin D levels. Although our estimation and conversion methods have been widely used in many reputable high-impact journals [51], there is still insufficient evidence to validate their accuracy, and they may introduce certain biases into the calculations [52]. Currently, IPD-based meta-analysis models have been proposed [53], and more data are becoming openly accessible to each researcher. IPD-based meta-analysis can provide results of higher evidential value, offering an excellent approach to producing more convincing conclusions in this field. Secondly, we cannot fully exclude the potential influence of seasonality and age on our study. Many studies have demonstrated that the season exerts a significant influence on an individual’s vitamin D status [54, 55]. The existing literature highlights the significant impact of seasonal fluctuations on vitamin D status, which is closely linked to solar (ultraviolet radiation B) UVB intensity [56, 57]. Thirdly, we contend that utilizing a uniform set of vitamin D level standards to assess vitamin D deficiency across different ethnicities lacks scientific rigor. Multiple studies have indicated there are variations in VDR gene polymorphisms across different ethnic groups [58, 59]. Therefore, a focused effort on establishing large-scale cohort studies specific to each ethnic group is crucial to investigate the threshold levels of vitamin D corresponding to different ethnicities. Such research aids in accurately assessing the extent of vitamin D deficiency among different ethnicities and formulating tailored intervention strategies. Additionally, it provides more precise reference values, enabling clinicians to offer personalized vitamin D supplementation recommendations to patients.

In the future, we anticipate the emergence of more extensive prospective clinical studies in this field, with the objective of elucidating the intricate association between vitamin D levels and comprehensive patient profiles during the ART process. By doing so, we aspire to accelerate the unraveling of the elusive threshold values pertaining to vitamin D, thereby enhancing our comprehension of its significance in optimizing ART outcomes.

Overall, the results of this review indicate that there is a correlation between vitamin D levels and both clinical pregnancy rates and live birth rates. Low vitamin D level does not influence ART outcomes in terms of biochemical pregnancy rate, ongoing pregnancy rate, miscarriage rate and implantation rate. Additionally, a nonlinear correlation was found between vitamin D levels and outcome measures when levels were below approximately 24 ng/L. Vitamin D levels showed little impact on the pooled OR below this threshold, while the degree of correlation increased beyond this threshold, suggesting that 24 ng/L may be a possible threshold of vitamin D concentration in assisted reproduction. More cohorts focusing on subgroups analysis and large size RCTs are needed in the future.

## Electronic Supplementary Material

Below is the link to the electronic supplementary material.

Supplementary Material 1

Supplementary Material 2

Supplementary Material 3

Supplementary Material 4

Supplementary Material 5

Supplementary Material 6

Supplementary Material 7

Supplementary Material 8

## Data Availability

All figures and tables including template data collection forms, data extracted from included studies, data used for all analyses are publicly available in the manuscript.
