# Supplementary material for: Association Between Vitamin D Level and Clinical Outcomes of Assisted Reproductive Treatment: A Systematic Review and Dose-Response Meta-Analysis
Source: Reprod Sci. 2024 May 22;32(5):1446–58. doi: 10.1007/s43032-024-01578-9 (PMC12041108; doi:10.1007/s43032-024-01578-9)
Supplement: Supplementary file 4 — Supplementary Material 4 [file 43032_2024_1578_MOESM4_ESM.docx]

**Supplementary file 1** Search strategy for databases

**COCHRANE**

ID Search

#1 MeSH descriptor: [Vitamin D] explode all trees

#2 MeSH descriptor: [25-Hydroxyvitamin D 2] explode all trees

#3 MeSH descriptor: [Ergocalciferols] explode all trees

#4 MeSH descriptor: [Cholecalciferol] explode all trees

#5 MeSH descriptor: [Calcitriol] explode all trees

#6 MeSH descriptor: [Reproductive Techniques, Assisted] explode all trees

#7 MeSH descriptor: [Fertilization in Vitro] explode all trees

#8 MeSH descriptor: [Sperm Injections, Intracytoplasmic] explode all trees

#9 (vitamin D) (Word variations have been searched)

#10 (25 Hydroxycalciferol) OR (25 Hydroxyvitamin D2) OR (25 Hydroxyergocalciferol) OR (Ercalcidiol) OR (25 Hydroxyvitamin D 2)

#11 (D2, Vitamin) OR (Vitamin D2) OR (Vitamin D 2) OR (Ergocalciferol) OR (Calciferols)

#12 (Vitamin D 3) OR (Vitamin D3) OR (Calciol) OR (Cholecalciferols)

#13 (Osteotriol) OR (Silkis) OR (Rocaltrol) OR (Calcijex) OR (KyraMed, Calcitriol)

#14 (Calcitriol KyraMed) OR (Sitriol) OR (MC1288) OR (MC 1288) OR (MC-1288)

#15 (Calcitriol Nefro) OR (Calcitriol-Nefro) OR (Bocatriol) OR (Tirocal) OR (Soltriol)

#16 (Renatriol) OR (Decostriol) OR (Colecalciferol) OR (Alpha Calcitriol) OR (alpha‐calcidol)

#17 #1 OR #2 OR #3 OR #4 OR #5 OR #9 OR #10 OR #11 OR #12 OR #13 OR #14 OR #15 OR #16

#18 (assisted reproductive technology) OR (Assisted Reproductive Technologies) OR (Technology, Assisted Reproductive) OR (Technologies, Assisted Reproductive) OR (Reproductive Technology, Assisted) (Word variations have been searched)

#19 (Reproductive Technologies, Assisted) OR (Techniques, Assisted Reproductive) OR (Reproductive Technique, Assisted) OR (Assisted Reproductive Techniques) OR (Assisted Reproductive Technic) (Word variations have been searched)

#20 (Technics, Assisted Reproductive) OR (Technique, Assisted Reproductive) OR (Assisted Reproductive Technics) OR (Reproductive Technic, Assisted) OR (Assisted Reproductive Technique) (Word variations have been searched)

#21 (Technic, Assisted Reproductive) OR (Reproductive Technics, Assisted) OR (in‐vitro fertilization) OR (Embryo) OR (Embryo Transfer) (Word variations have been searched)

#22 (Fertilization, Test-Tube) OR (Test-Tube Fertilization) OR (Fertilizations in Vitro) OR (In Vitro Fertilizations) OR (Test Tube Fertilization) (Word variations have been searched)

#23 (Fertilizations, Test-Tube) OR (Test-Tube Fertilizations) OR (In Vitro Fertilization) OR (Test-Tube Baby) OR (Babies, Test-Tube) (Word variations have been searched)

#24 (Test Tube Babies) OR (Baby, Test-Tube) OR (Test-Tube Babies) (Word variations have been searched)

#25 (Intracytoplasmic Sperm Injection) OR (Intracytoplasmic Sperm Injections) OR (Injections, Sperm, Intracytoplasmic) OR (Injections, Intracytoplasmic Sperm) OR (ICSI) (Word variations have been searched)

#26 (Sperm Injection, Intracytoplasmic) OR (Injection, Intracytoplasmic Sperm) (Word variations have been searched)

#27 #18 OR #19 OR #20 OR #21

#28 #22 OR #23 OR #24

#29 #25 OR #26

#30 #6 OR #27

#31 #7 OR #28

#32 #8 OR #29

#33 #30 OR #31 OR #32

#34 #33 AND #17 **184**

**PUBMED**

#1 Vitamin D

#2 25-Hydroxyvitamin D

#3 Ergocalciferols

#4 Cholecalciferol

#5 Calcitriol

#6 Reproductive Techniques, Assisted

#7 Fertilization in Vitro

#8 Sperm Injections, Intracytoplasmic

#9 (25 Hydroxycalciferol) OR (25 Hydroxyvitamin D2) OR (25 Hydroxyergocalciferol) OR (Ercalcidiol) OR (25 Hydroxyvitamin D 2)

#10 (D2, Vitamin) OR (Vitamin D2) OR (Vitamin D 2) OR (Ergocalciferol) OR (Calciferols)

#11 (Vitamin D 3) OR (Vitamin D3) OR (Calciol) OR (Cholecalciferols)

#12 (Osteotriol) OR (Silkis) OR (Rocaltrol) OR (Calcijex) OR (KyraMed, Calcitriol)

#13 (Calcitriol KyraMed) OR (Sitriol) OR (MC1288) OR (MC 1288) OR (MC-1288)

#14 (Calcitriol Nefro) OR (Calcitriol-Nefro) OR (Bocatriol) OR (Tirocal) OR (Soltriol)

#15 (Renatriol) OR (Decostriol) OR (Colecalciferol) OR (Alpha Calcitriol) OR (alpha‐calcidol)

#16 #1 OR #2 OR #3 OR #4 OR #5 OR #9 OR #10 OR #11 OR #12 OR #13 OR #14 OR #15

#17 (assisted reproductive technology) OR (Assisted Reproductive Technologies) OR (Technology, Assisted Reproductive) OR (Technologies, Assisted Reproductive) OR (Reproductive Technology, Assisted)

#18 (Reproductive Technologies, Assisted) OR (Techniques, Assisted Reproductive) OR (Reproductive Technique, Assisted) OR (Assisted Reproductive Techniques) OR (Assisted Reproductive Technic)

#19 (Technics, Assisted Reproductive) OR (Technique, Assisted Reproductive) OR (Assisted Reproductive Technics) OR (Reproductive Technic, Assisted) OR (Assisted Reproductive Technique)

#20 (Technic, Assisted Reproductive) OR (Reproductive Technics, Assisted) OR (in‐vitro fertilization)

#21 (Fertilization, Test-Tube) OR (Test-Tube Fertilization) OR (Fertilizations in Vitro) OR (In Vitro Fertilizations) OR (Test Tube Fertilization)

#22 (Fertilizations, Test-Tube) OR (Test-Tube Fertilizations) OR (In Vitro Fertilization) OR (Test-Tube Baby) OR (Babies, Test-Tube)

#23 (Test Tube Babies) OR (Baby, Test-Tube) OR (Test-Tube Babies)

#24 (Intracytoplasmic Sperm Injection) OR (Intracytoplasmic Sperm Injections) OR (Injections, Sperm, Intracytoplasmic) OR (Injections, Intracytoplasmic Sperm) OR (ICSI)

#25 (Sperm Injection, Intracytoplasmic) OR (Injection, Intracytoplasmic Sperm)

#26 #17 OR #18 OR #19 OR #20

#27 #21 OR #22 OR #23

#28 #24 OR #25

#29 #6 OR #26

#30 #7 OR #27

#31 #8 OR #28

#32 #29 OR #30 OR #31

#33 #32 AND #16 **228**

**Embase**

No. Query

#1. 'vitamin d'/exp OR 'vitamin d'

#2. '25-hydroxyvitamin d 2'/exp OR '25-hydroxyvitamin d 2

#3. 'ergocalciferols'/exp OR 'ergocalciferols’

#4. 'cholecalciferol'/exp OR 'cholecalciferol'

#5. 'calcitriol'/exp OR 'calcitriol'

#6. 'reproductive techniques, assisted'/exp OR 'reproductive techniques, assisted'

#7. 'fertilization in vitro'/exp OR 'fertilization in vitro'

#8. 'sperm injections, intracytoplasmic'/exp OR 'sperm injections, intracytoplasmic'

#9. '25 hydroxycalciferol' OR '25 hydroxyvitamin d2' OR '25 hydroxyergocalciferol' OR 'ercalcidiol' OR '25 hydroxyvitamin d 2'

#10. 'd2, vitamin' OR 'vitamin d2' OR 'vitamin d 2' OR 'ergocalciferol' OR 'calciferols'

#11. 'vitamin d 3' OR 'vitamin d3' OR 'calciol' OR 'cholecalciferols'

#12. 'osteotriol' OR 'silkis' OR 'rocaltrol' OR 'calcijex' OR 'kyramed, calcitriol'

#13. 'calcitriol kyramed' OR 'sitriol' OR 'mc1288' OR 'mc 1288' OR 'mc-1288'

#14. 'calcitriol nefro' OR 'calcitriol-nefro' OR 'bocatriol' OR 'tirocal' OR 'soltriol'

#15. 'renatriol' OR 'decostriol' OR 'colecalciferol' OR 'alpha calcitriol' OR 'alpha‐calcidol'

#16. #1 OR #2 OR #3 OR #4 OR #5 OR #9 OR #10 OR #11 OR #12 OR #13 OR #14 OR #15

#17. 'assisted reproductive technology' OR 'assisted reproductive technologies' OR 'technology, assisted reproductive' OR 'technologies, assisted reproductive' OR 'reproductive technology, assisted'

#18. 'reproductive technologies, assisted' OR 'techniques, assisted reproductive' OR 'reproductive technique, assisted' OR 'assisted reproductive techniques' OR 'assisted reproductive technic'

#19. 'technics, assisted reproductive' OR 'technique, assisted reproductive' OR 'assisted reproductive technics' OR 'reproductive technic, assisted' OR 'assisted reproductive technique'

#20. 'technic, assisted reproductive' OR 'reproductive technics, assisted' OR 'in‐vitro fertilization'

#21. 'fertilization, test-tube' OR 'test-tube fertilization' OR 'fertilizations in vitro' OR 'in vitro fertilizations' OR 'test tube fertilization'

#22. 'fertilizations, test-tube' OR 'test-tube fertilizations' OR 'in vitro fertilization' OR 'test-tube baby' OR 'babies, test-tube'

#23. 'test tube babies' OR 'baby, test-tube' OR 'test-tube babies'

#24. 'intracytoplasmic sperm injection' OR 'intracytoplasmic sperm injections' OR 'injections, sperm, intracytoplasmic' OR 'injections, intracytoplasmic sperm' OR 'icsi'

#25. 'sperm injection, intracytoplasmic' OR 'injection, intracytoplasmic sperm'

#26. #17 OR #18 OR #19 OR #20

#27. #21 OR #22 OR #23

#28. #24 OR #25

#29. #6 OR #26

#30. #7 OR #27

#31. #8 OR #28

#32. #29 OR #30 OR #31

#33. #32 AND #16 **657**

**WEB OF SCIENCE**

# 检索:

1: TS=(Vitamin D)

2: TS=(25-Hydroxyvitamin D )

3: TS=(Ergocalciferols )

4: TS=(Cholecalciferol )

5: TS=(Calcitriol )

6: TS=(Reproductive Techniques, Assisted )

7: TS=(Fertilization in Vitro)

8: TS=(Sperm Injections, Intracytoplasmic )

9: TS=(25 Hydroxycalciferol) OR TS=(25 Hydroxyvitamin D2) OR TS=(25 Hydroxyergocalciferol) OR TS=(Ercalcidiol) OR TS=(25 Hydroxyvitamin D 2)

10: TS= (D2, Vitamin) OR TS= (Vitamin D2) OR TS= (Vitamin D 2) OR TS= (Ergocalciferol) OR TS= (Calciferols)

11: TS= (Vitamin D 3) OR TS= (Vitamin D3) OR TS= (Calciol) OR TS= (Cholecalciferols)

12: TS=(Osteotriol) OR TS=(Silkis) OR TS=(Rocaltrol) OR TS=(Calcijex) OR TS=(KyraMed, Calcitriol)

13: TS=(Calcitriol KyraMed) OR TS=(Sitriol) OR TS=(MC1288) OR TS=(MC 1288) OR TS=(MC-1288)

14: TS=(Calcitriol Nefro) OR TS=(Calcitriol-Nefro) OR TS=(Bocatriol) OR TS=(Tirocal) OR TS=(Soltriol)

15: TS=(Renatriol) OR TS=(Decostriol) OR TS=(Colecalciferol) OR TS=(Alpha Calcitriol) OR TS=(alpha‐calcidol)

#16 #1 OR #2 OR #3 OR #4 OR #5 OR #9 OR #10 OR #11 OR #12 OR #13 OR #14 OR #15

17: TS=(assisted reproductive technology) OR TS=(Assisted Reproductive Technologies) OR TS=(Technology, Assisted Reproductive) OR TS=(Technologies, Assisted Reproductive) OR TS=(Reproductive Technology, Assisted)

18: TS=(Reproductive Technologies, Assisted) OR TS=(Techniques, Assisted Reproductive) OR TS=(Reproductive Technique, Assisted) OR TS=(Assisted Reproductive Techniques) OR TS=(Assisted Reproductive Technic)

19: TS=(Technics, Assisted Reproductive) OR TS=(Technique, Assisted Reproductive) OR TS=(Assisted Reproductive Technics) OR TS=(Reproductive Technic, Assisted) OR TS=(Assisted Reproductive Technique)

20: TS=(Technic, Assisted Reproductive) OR TS=(Reproductive Technics, Assisted) OR TS=(in‐vitro fertilization)

21: TS=(Fertilization, Test-Tube) OR TS=(Test-Tube Fertilization) OR TS=(Fertilizations in Vitro) OR TS=(In Vitro Fertilizations) OR TS=(Test Tube Fertilization)

22: TS=(Fertilizations, Test-Tube) OR TS=(Test-Tube Fertilizations) OR TS=(In Vitro Fertilization) OR TS=(Test-Tube Baby) OR TS=(Babies, Test-Tube)

23: TS=(Test Tube Babies) OR TS=(Baby, Test-Tube) OR TS=(Test-Tube Babies)

24: TS=(Intracytoplasmic Sperm Injection) OR TS=(Intracytoplasmic Sperm Injections) OR TS=(Injections, Sperm, Intracytoplasmic) OR TS=(Injections, Intracytoplasmic Sperm) OR TS=(ICSI)

25: TS=(Sperm Injection, Intracytoplasmic) OR TS=(Injection, Intracytoplasmic Sperm)

26: #17 OR #18 OR #19 OR #20

27: #21 OR #22 OR #23

28: #24 OR #25

29: #6 OR #26

30: #7 OR #27

31: #8 OR #28

32: #29 OR #30 OR #31

33: #32 AND #16  **441**

**ClinicalTrails.gov**

(Vitamin D) OR (25-Hydroxyvitamin D) OR (Ergocalciferols) OR (Cholecalciferol) OR (Calcitriol)] AND [(assisted reproductive technology) OR (Fertilization in Vitro) OR (Sperm Injections, Intracytoplasmic) OR (Test Tube Babies) OR (IVF) OR (ICSI) **26**
