# Supplementary material for: Association Between Vitamin D Level and Clinical Outcomes of Assisted Reproductive Treatment: A Systematic Review and Dose-Response Meta-Analysis
Source: Reprod Sci. 2024 May 22;32(5):1446–58. doi: 10.1007/s43032-024-01578-9 (PMC12041108; doi:10.1007/s43032-024-01578-9)
Supplement: Supplementary file 5 — Supplementary Material 5 [file 43032_2024_1578_MOESM5_ESM.docx]

**Supplementary file 2** Quality assessment of included studies based on Newcastle-Ottawa Scale.

| Study | Selection | | | | Comparability | Outcome | | | Score |  |
| --- | --- | --- | --- | --- | --- | --- | --- | --- | --- | --- |
|  | Represe ntativen ess of the expose d  cohort | Selection of the non- exposed cohort | Ascerta inment of the exposur e | Demonstration that outcomes of interest was not present at start of study | Comparability of cohorts on the basis of the design or analysis | Asses sment of outco me | Was follow-up long enough for outcomes  to occur | Adequa cy of follow- up of cohorts | |  |
| Georgios.M Anifandis,2010 |  | * | * |  | * | * | * | * | 6 |  |
| B. Rudick,2012 | * | * | * |  | ** | * | * | * | 8 |  |
| Garbedian 2013 |  | * | * |  | * | * | * | * | 6 |  |
| B. Rudick,2014 | * | * | * |  | * | * | * | * | 7 |  |
| Fabris, 2014 | * | * | * |  | * | * | * | * | 7 |  |
| Alessio Paffoni,2014 | * | * | * |  | ** | * | * | * | 8 |  |
| Nikolaos.P.Polyzos,2014 | * | * | * |  | * | * | * | * | 7 |  |
| Firouzabadi 2014 |  | * | * |  | * | * | * | * | 6 |  |
| Jason.M. Franasiak,2015 |  | * | * |  | * | * | * | * | 6 |  |
| Arne van de Vijver, 2016 | * | * | * |  | * | * | * | * | 7 |  |
| Abadia 2016 |  | * | * |  | * | * | * | * | 6 |  |
| Banker 2017 | * | * | * |  |  | * | * | * | 6 |  |
| Fabris 2017 |  | * | * |  | * | * | * | * | 6 |  |
| Ciepiela 2018 | * | * | * |  | * | * | * | * | 7 |  |
| K Y Ko 2019 |  | * | * |  | * | * | * | * | 6 |  |
| Xuemei Liu 2019 |  | * | * | * |  | * | * | * | 6 |  |
| Chu 2019 | * | * | * |  |  | * | * | * | 7 |  |
| Walz,N.L, 2020 |  | * | * |  | * | * | * | * | 6 |  |
| Cai,S,2021 | * | * | * |  | * | * | * | * | 7 |  |
| Neysanian 2021 |  | * | * |  | * | * | * | * | 6 |  |
| Muyayalo 2022 |  | * | * |  | * | * | * | * | 6 |  |
| Yu Z,2022 | * | * | * |  | * | * | * | * | 7 |  |
| Hasan, H. A.,2023 |  | * | * |  | * | * | * | * | 6 |  |
