# Supplementary material for: Association Between Vitamin D Level and Clinical Outcomes of Assisted Reproductive Treatment: A Systematic Review and Dose-Response Meta-Analysis
Source: Reprod Sci. 2024 May 22;32(5):1446–58. doi: 10.1007/s43032-024-01578-9 (PMC12041108; doi:10.1007/s43032-024-01578-9)

**Supplementary file 3** Relevant Statistical Figures

**Supplementary Figure 3.1|** Meta-analysis of studies reporting biochemical pregnancy in sufficient vitamin D(≥30ng/ml) + insufficient vitamin D(20-30ng/ml) and deficient vitamin D(<20ng/ml). CI, confidence interval.


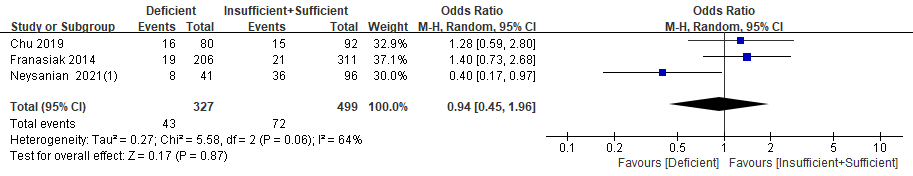


**Supplementary Figure 3.2|** Meta-analysis of studies reporting biochemical pregnancy in sufficient vitamin D(≥30ng/ml) and deficient vitamin D(<20ng/ml) + insufficient vitamin D(20-30ng/ml). CI, confidence interval.


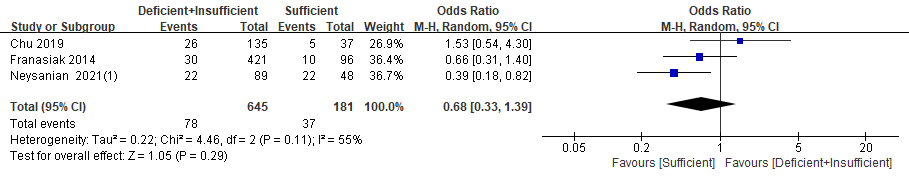


**Supplementary Figure 3.3|** Meta-analysis of studies reporting LBR in sufficient vitamin D(≥30ng/ml) + insufficient vitamin D(20-30ng/ml) and deficient vitamin D(<20ng/ml). CI, confidence interval.


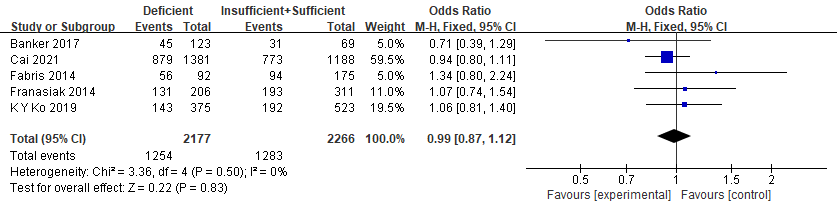


**Supplementary Figure 3.4|** Meta-analysis of studies reporting LBR in sufficient vitamin D(≥30ng/ml) and deficient vitamin D(<20ng/ml) + insufficient vitamin D(20-30ng/ml). CI, confidence interval.


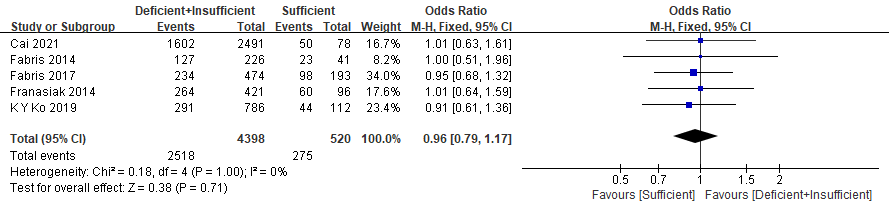


**Supplementary Figure 3.5|** Meta-analysis of studies reporting miscarriage in sufficient vitamin D(≥30ng/ml) + insufficient vitamin D(20-30ng/ml) and deficient vitamin D(<20ng/ml). CI, confidence interval.


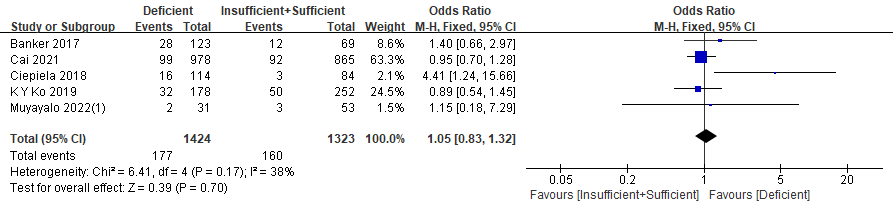


**Supplementary Figure 3.6|** Meta-analysis of studies reporting miscarriage in sufficient vitamin D(≥30ng/ml) and deficient vitamin D(<20ng/ml) + insufficient vitamin D(20-30ng/ml). CI, confidence interval.


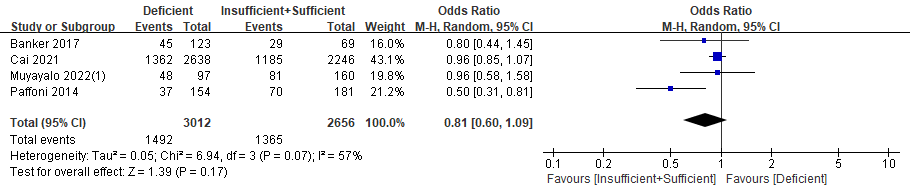


**Supplementary Figure 3.7|** Meta-analysis of studies reporting LBR in sufficient vitamin D(≥30ng/ml) + insufficient vitamin D(20-30ng/ml) and deficient vitamin D(<20ng/ml). CI, confidence interval.


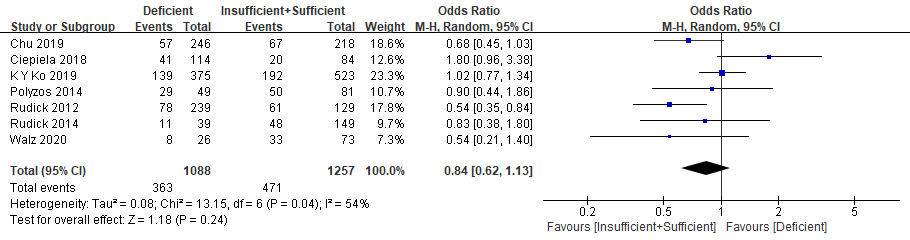


**Supplementary Figure 3.8|** Meta-analysis of studies reporting LBR in sufficient vitamin D(≥30ng/ml) and deficient vitamin D(<20ng/ml) + insufficient vitamin D(20-30ng/ml). CI, confidence interval.


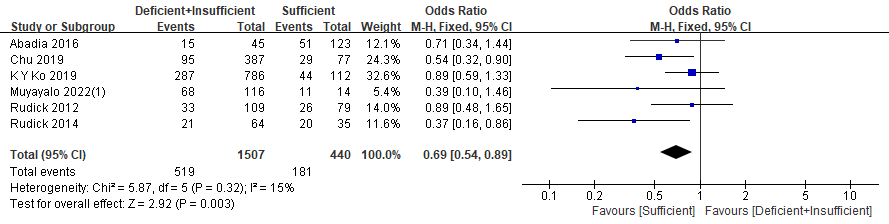


**Supplementary Figure 3.9|** Meta-analysis of studies reporting IR in sufficient vitamin D(≥30ng/ml) + insufficient vitamin D(20-30ng/ml) and deficient vitamin D(<20ng/ml). CI, confidence interval.


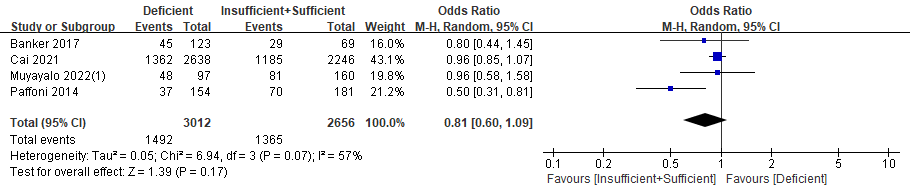


**Supplementary Figure 3.10|**Meta-analysis of studies reporting IR in sufficient vitamin D(≥30ng/ml) and deficient vitamin D(<20ng/ml) + insufficient vitamin D(20-30ng/ml). CI, confidence interval.


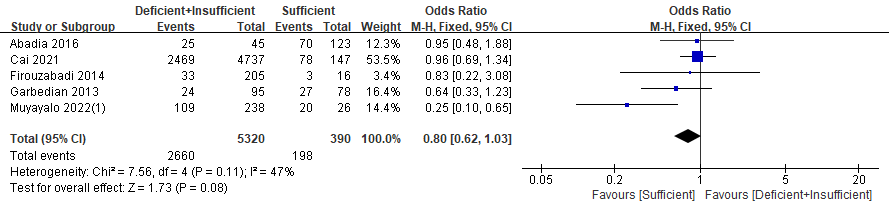

Supplement: Supplementary file 6 — Supplementary Material 6 [file 43032_2024_1578_MOESM6_ESM.docx]
