# Supplementary material for: Association Between Vitamin D Level and Clinical Outcomes of Assisted Reproductive Treatment: A Systematic Review and Dose-Response Meta-Analysis
Source: Reprod Sci. 2024 May 22;32(5):1446–58. doi: 10.1007/s43032-024-01578-9 (PMC12041108; doi:10.1007/s43032-024-01578-9)
Supplement: Supplementary file 7 — Supplementary Material 7 [file 43032_2024_1578_MOESM7_ESM.docx]

**Supplementary file 4** Publication bias

**Supplementary Figure 4.1|** Publication bias on Deficient vitamin D level versus Insufficient + Sufficient vitamin D level, using Funnel Plot

**Supplementary Figure 4.2|** Publication bias on Deficient vitamin D level versus Insufficient + Sufficient vitamin D level, using Egger’s Test

**Supplementary Figure 4.3|** Publication bias on Deficient + Insufficient vitamin D level versus Sufficient vitamin D level, using Funnel Plot

**Supplementary Figure 4.4|** Publication bias on Deficient + Insufficient vitamin D level versus Sufficient vitamin D level, using Egger’s Test
