# Supplementary material for: Association Between Vitamin D Level and Clinical Outcomes of Assisted Reproductive Treatment: A Systematic Review and Dose-Response Meta-Analysis
Source: Reprod Sci. 2024 May 22;32(5):1446–58. doi: 10.1007/s43032-024-01578-9 (PMC12041108; doi:10.1007/s43032-024-01578-9)

**Supplementary file 5** Subgroup Statistical Figures and Analysis

**Source of Oocyte**

**Supplementary Figure 5.1.1|** Subgroup meta-analysis of studies reporting clinical pregnancy rate in sufficient vitamin D(≥30ng/ml) + insufficient vitamin D(20-30ng/ml) and deficient vitamin D(<20ng/ml), group divided by source of oocyte. CI, confidence interval.


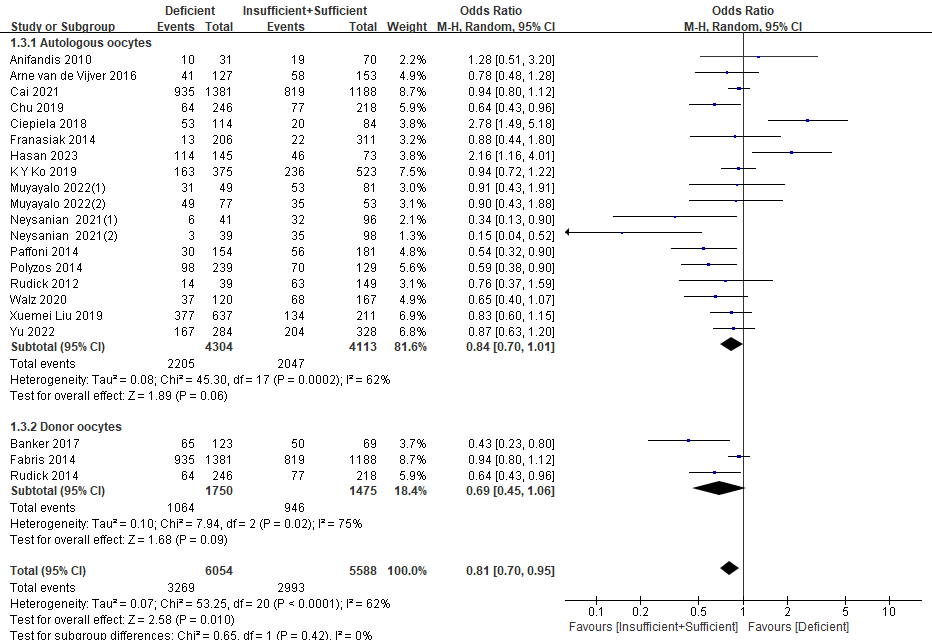


**Supplementary Figure 5.1.2|** Subgroup meta-analysis of studies reporting clinical pregnancy rate in sufficient vitamin D(≥30ng/ml) and deficient vitamin D(<20ng/ml) + insufficient vitamin D(20-30ng/ml), group divided by source of oocyte. CI, confidence interval.


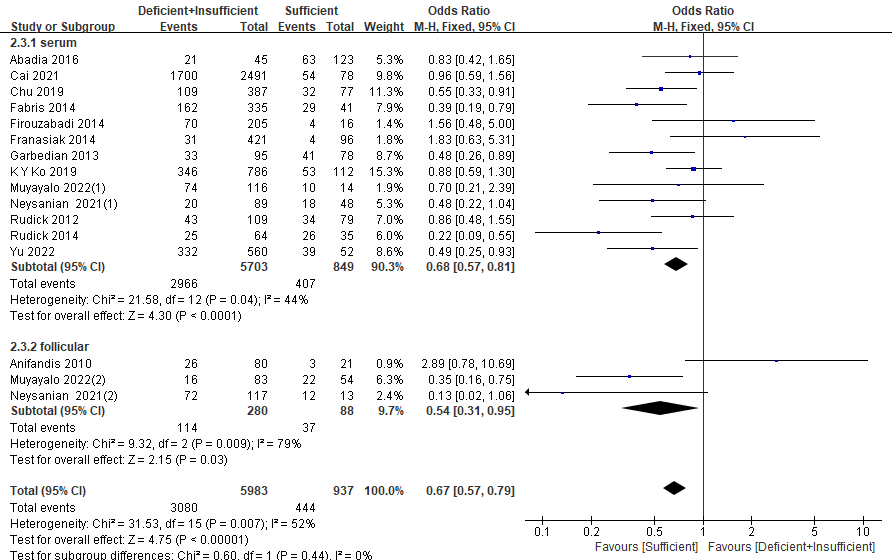


**Source of Vitamin D.**

**Supplementary Figure 5.2.1|** Subgroup meta-analysis of studies reporting clinical pregnancy rate in sufficient vitamin D(≥30ng/ml) + insufficient vitamin D(20-30ng/ml) and deficient vitamin D(<20ng/ml), group divided by source of vitamin D. CI, confidence interval.


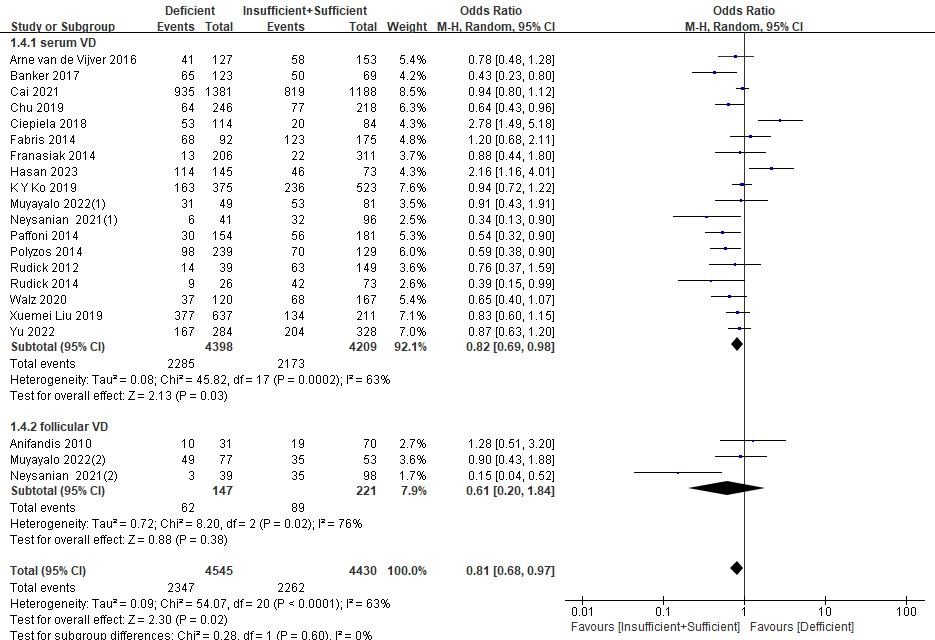


**Supplementary Figure 5.2.2|** Subgroup meta-analysis of studies reporting clinical pregnancy rate in sufficient vitamin D(≥30ng/ml) and deficient vitamin D(<20ng/ml) + insufficient vitamin D(20-30ng/ml), group divided by source of vitamin D. CI, confidence interval.


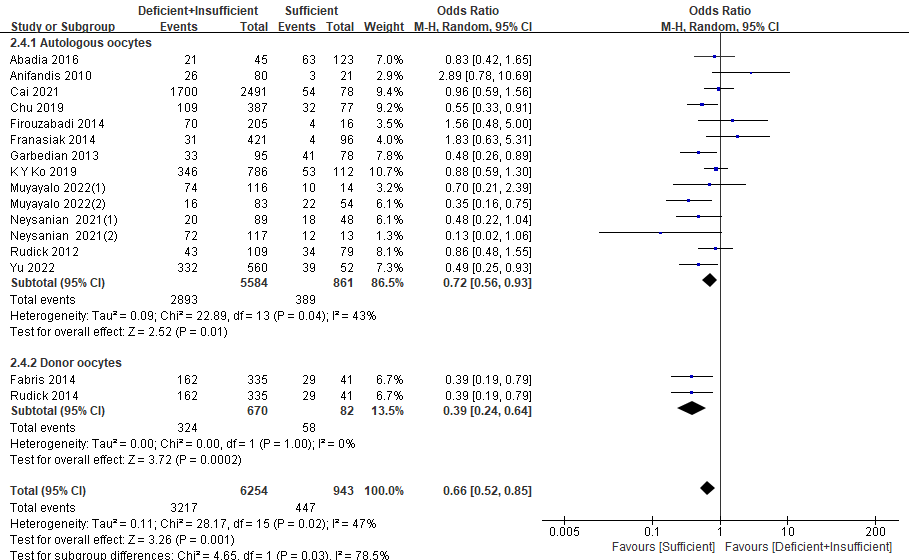


**Race**

**Supplementary Figure 5.3.1|** Subgroup meta-analysis of studies reporting clinical pregnancy rate in sufficient vitamin D(≥30ng/ml) + insufficient vitamin D(20-30ng/ml) and deficient vitamin D(<20ng/ml), group divided by race. CI, confidence interval.


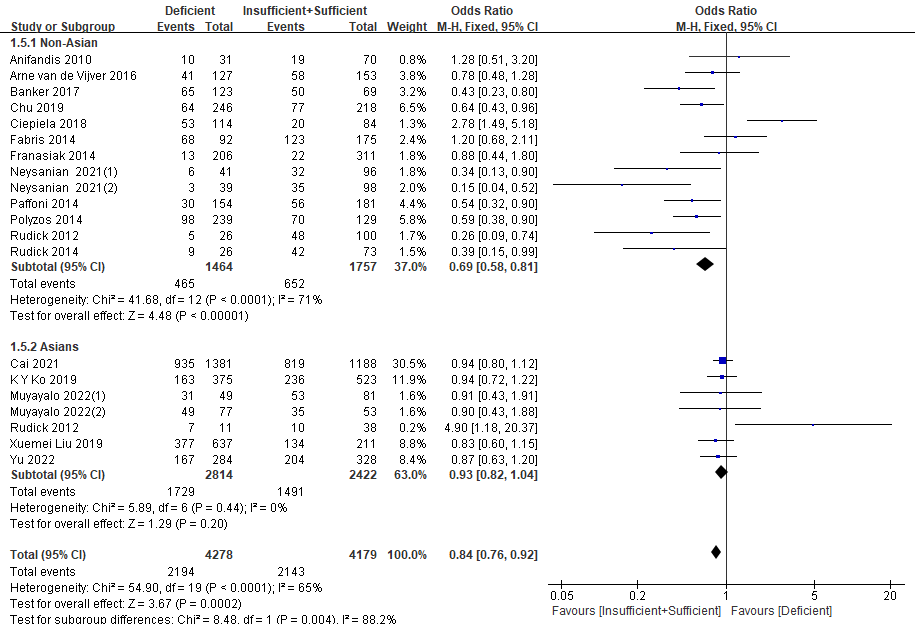


**Supplementary Figure 5.3.2|** Subgroup meta-analysis of studies reporting clinical pregnancy rate in sufficient vitamin D(≥30ng/ml) and deficient vitamin D(<20ng/ml) + insufficient vitamin D(20-30ng/ml), group divided by race. CI, confidence interval.


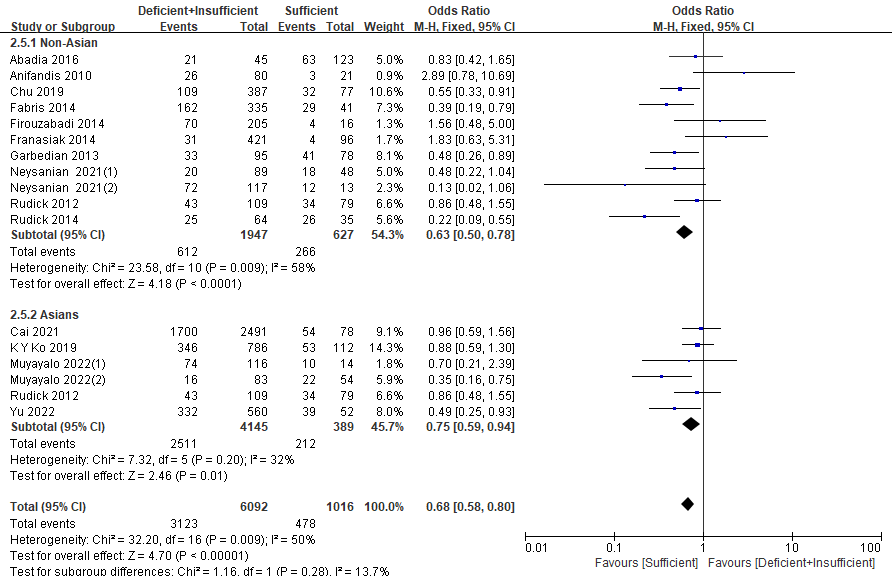


**Study Design**

**Supplementary Figure 5.4.1|** Subgroup meta-analysis of studies reporting clinical pregnancy rate in sufficient vitamin D(≥30ng/ml) + insufficient vitamin D(20-30ng/ml) and deficient vitamin D(<20ng/ml), group divided by study design. CI, confidence interval.


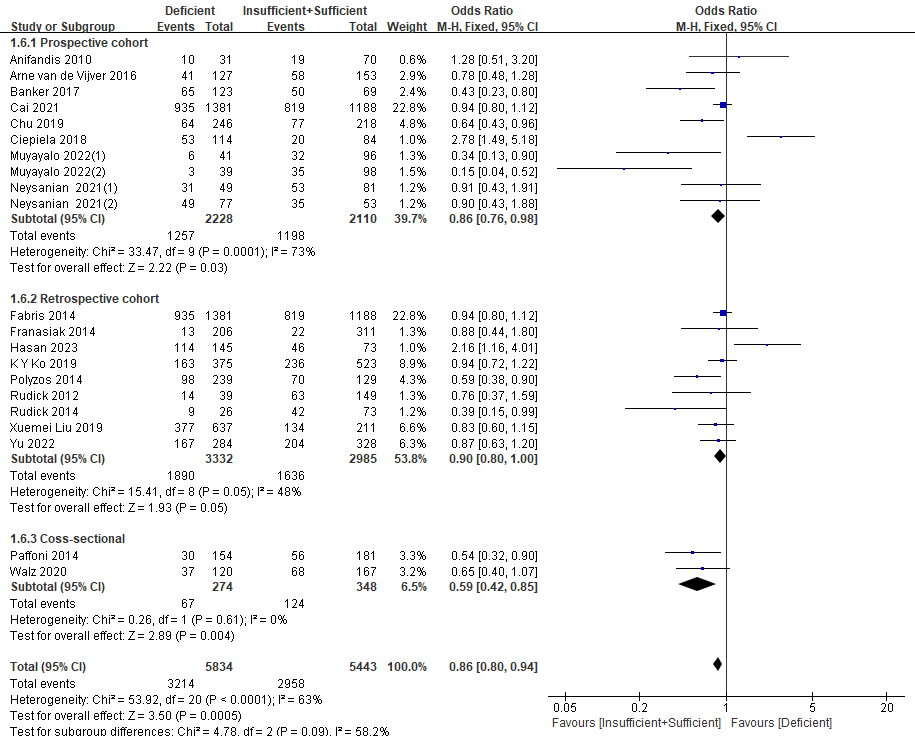


**Supplementary Figure 5.4.2|** Subgroup meta-analysis of studies reporting clinical pregnancy rate in sufficient vitamin D(≥30ng/ml) and deficient vitamin D(<20ng/ml) + insufficient vitamin D(20-30ng/ml), group divided by study design. CI, confidence interval.


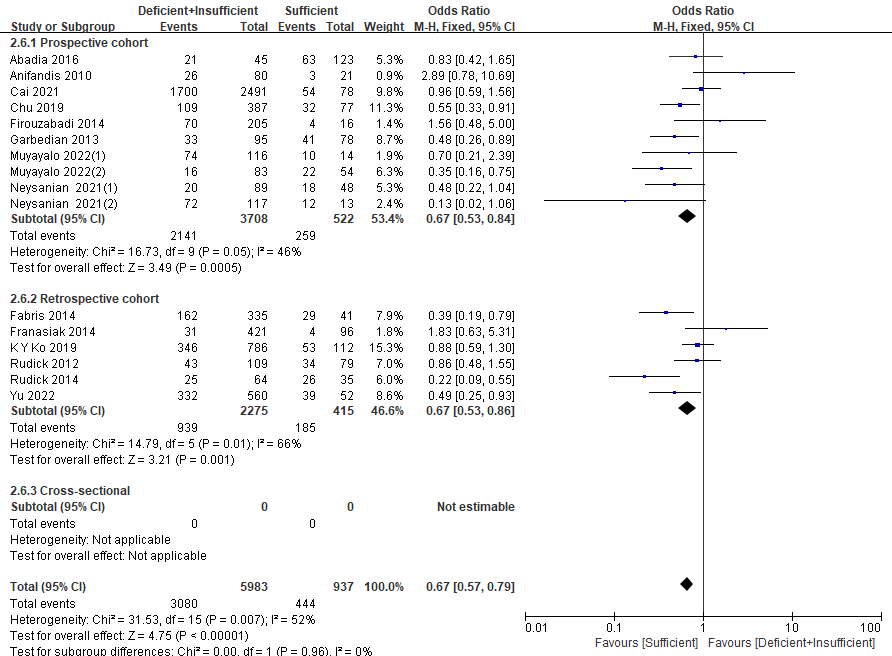


**Method of Vitamin D Assessment**

**Supplementary Figure 5.5.1|** Subgroup meta-analysis of studies reporting clinical pregnancy rate in sufficient vitamin D(≥30ng/ml) + insufficient vitamin D(20-30ng/ml) and deficient vitamin D(<20ng/ml), group divided by method of vitamin D assessment. CI, confidence interval.


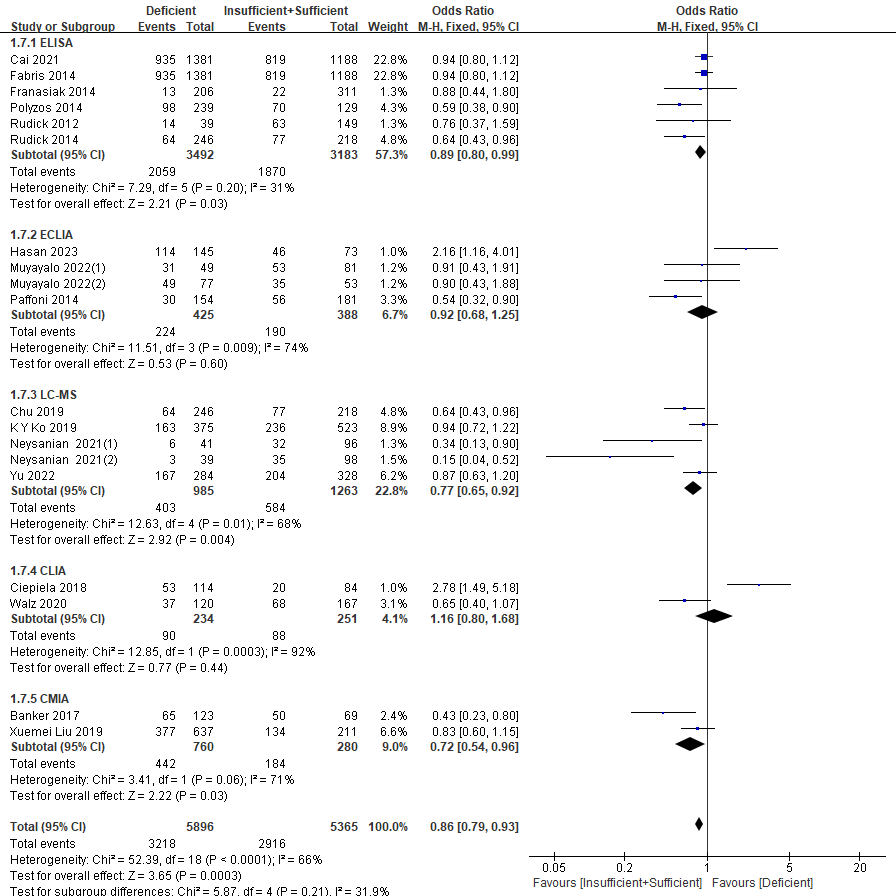


**Supplementary Figure 5.5.2|** Subgroup meta-analysis of studies reporting clinical pregnancy rate in sufficient vitamin D(≥30ng/ml) and deficient vitamin D(<20ng/ml) + insufficient vitamin D(20-30ng/ml), group divided by method of vitamin D assessment. CI, confidence interval.


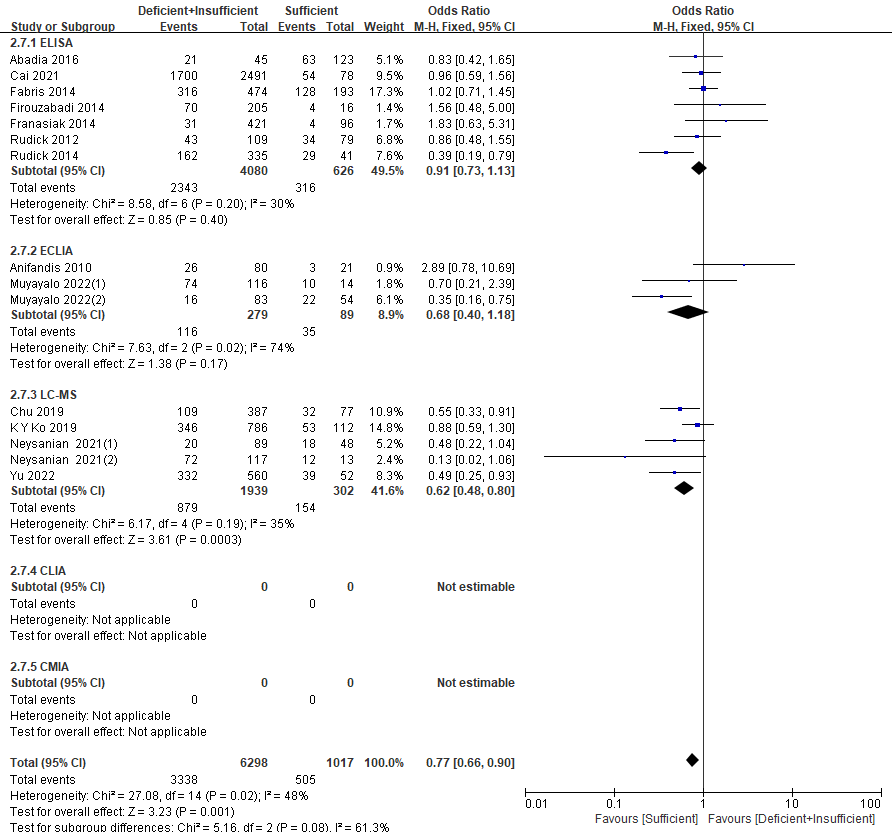


**Time of Sample Collection**

**Supplementary Figure 5.6.1|** Subgroup meta-analysis of studies reporting clinical pregnancy rate in sufficient vitamin D(≥30ng/ml) + insufficient vitamin D(20-30ng/ml) and deficient vitamin D(<20ng/ml), group divided by time of sample collection. CI, confidence interval.


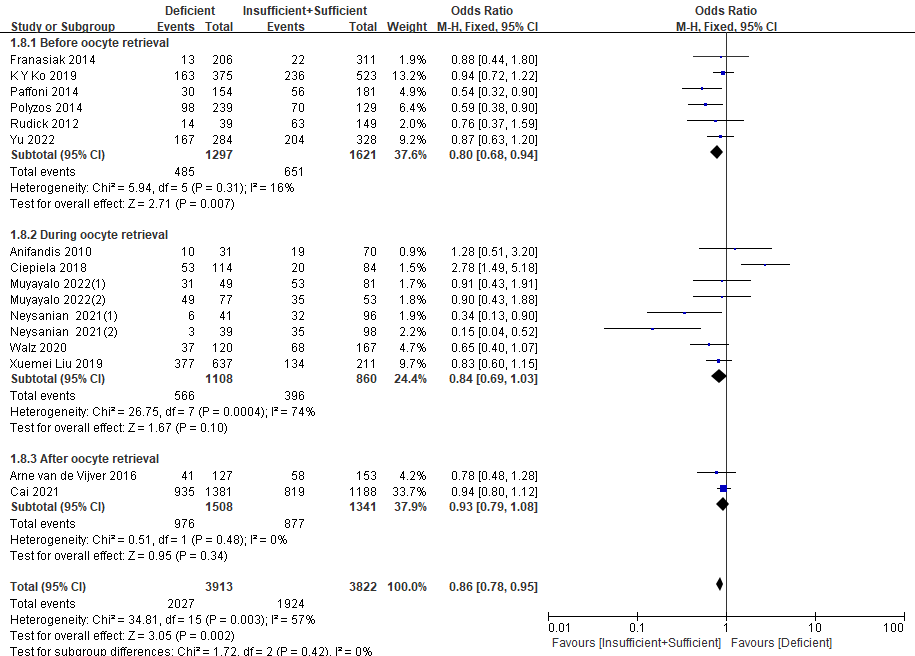


**Supplementary Figure 5.6.2|** Subgroup meta-analysis of studies reporting clinical pregnancy rate in sufficient vitamin D(≥30ng/ml) and deficient vitamin D(<20ng/ml) + insufficient vitamin D(20-30ng/ml), group divided by time of sample collection. CI, confidence interval.


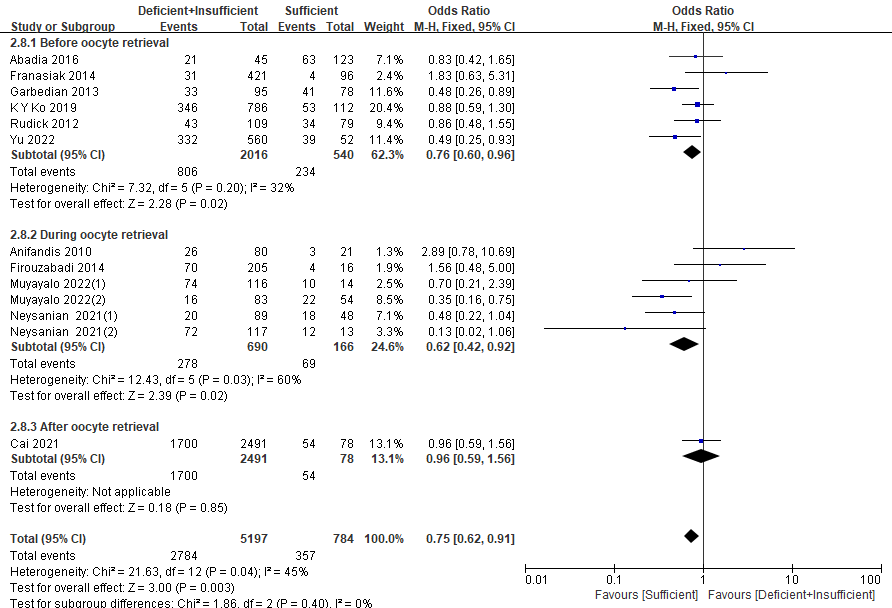

Supplement: Supplementary file 8 — Supplementary Material 8 [file 43032_2024_1578_MOESM8_ESM.docx]
